# Anti‐Aging Potential of Callus Culture: Mechanisms, Efficacy and Future Trends

**DOI:** 10.1111/jocd.71066

**Published:** 2026-08-28

**Authors:** Amir Mohammad Sharafi, Ali Khajeei, Maryam Hasan Zadeh Navroodi, Soheila Mokari, Seydeh Halimeh Najafi, Mohaddeseh Argha, Faezeh Talaei Shahmirzadi, Parina Asgharian, Tooba Gholikhani

**Affiliations:** ^1^ Faculty of Pharmacy Tabriz University of Medical Sciences Tabriz Iran; ^2^ Department of Pharmaceutics, Faculty of Pharmacy Tehran University of Medical Sciences Tehran Iran; ^3^ Drug and Natural Products Growth Center of IAU Amoli Branch Amol Iran; ^4^ Pharmacognosy, Faculty of Pharmacy Tabriz University of Medical Sciences Tabriz Iran; ^5^ Department of Pharmacy, Am.C. Islamic Azad University Amol Iran

**Keywords:** anti‐aging, antioxidants, callus culture, collagen, skin, skincare

## Abstract

**Background:**

Callus culture technology revolutionizes skin antiaging research, offering a sustainable and efficient method for producing bioactive compounds with potent geroprotective properties. This plant tissue and cell culture technique enables the controlled biosynthesis of beneficial phytochemicals while mitigating the variability and contamination risks inherent in traditional plant sources. By manipulating phytohormonal pathways, researchers can increase the yield of secondary metabolites that possess therapeutic and cosmetic efficacy, aligning with ethical standards of environmental stewardship.

**Methods:**

A thorough literature search was carried out to assess the anti‐aging potential of callus culture using the search terms (callus culture OR plant stem cells) AND (anti‐aging OR skin aging OR photoaging) AND (skin OR skincare) AND (bioactive compounds OR phytochemicals OR antioxidants). Additional searches included (mechanisms OR collagen OR anti‐inflammatory OR wound healing) AND (cosmetic OR cosmeceutical OR skin rejuvenation). Relevant studies were also identified by screening the reference lists of the retrieved articles.

**Results:**

Numerous studies have demonstrated the efficacy of callus cultures derived from various plant species, such as *Malus domestica* (apple), *Oryza sativa* (rice), *Centella asiatica* , and *Aster yomena*, in generating phytochemicals rich in antioxidants and anti‐inflammatory agents. These compounds promote skin health and combat oxidative stress. In vitro cultures have shown the ability to increase keratinocyte proliferation, stimulate collagen synthesis, inhibit elastase activity, and reduce the levels of inflammatory markers associated with skin aging. Furthermore, the callus culture extracts mitigate the adverse effects of oxidative stress and inflammation, major contributors to skin aging. This renders them invaluable sources of geroprotective agents suitable for cosmeceutical and pharmaceutical applications. The growing consumer demand for natural ingredients has led to a notable market shift toward the use of botanical preparations for anti‐aging solutions.

**Conclusion:**

In conclusion, the integration of callus culture techniques offers a novel approach for the development of antiaging skincare products. By leveraging the inherent ability of plant cells to synthesize bioactive metabolites, researchers and cosmetic scientists can create effective and sustainable formulations that meet the demand for natural skincare solutions.

**Discussion:**

It should be emphasized that the evidence presented in this review shows mixed conclusions: the majority of reported effects derive from acellular biochemical assays (e.g., DPPH, ABTS, ORAC) and cell‐based studies in keratinocytes and fibroblasts, whereas controlled human clinical data remain limited to a small number of studies. Accordingly, the geroprotective claims presented here should be interpreted in light of this evidence hierarchy, and the conclusions are framed as promising but preliminary pending larger clinical validation.

## 1 Introduction

The human affinity for botanicals in health and beauty products stretches back millennia, embedding their use deeply into the traditions of past and present civilizations. Plant‐derived ingredients, once the sole domain of natural colorants, calming balms, and aromatic oils, have evolved their cosmetic origins to become a cornerstone of the modern cosmeceutical landscape [1, 2]. This burgeoning popularity of these products brings with it a challenging landscape to navigate, including the risks of adverse effects, the lack of standardized regulations, and the need for rigorous safety and effectiveness evaluations [3]. The confluence of scientific innovation and consumer demand has placed botanical ingredients at the forefront of the cosmeceutical industry, which increasingly uses plant extracts for their health‐promoting and beautifying properties. Among the herbal compounds, polyphenols and flavonoids, lauded for their antioxidant properties, have become prominent fixtures in skincare formulations [2, 4]. The application of bioactive extracts or phytochemicals derived from a variety of botanicals in cosmetics serves a dual function: caring for the body and serving as a key element in cosmetic formulations. The benefits of plant extracts in skincare products include their antioxidant activity, tyrosinase inhibition effect, and antimicrobial activity [5]. This trend extends beyond topical applications, as the use of botanical preparations in anti‐aging products has experienced a significant surge. With consumers increasingly gravitating toward natural ingredients, 50% of British consumers are actively seeking cosmetics formulated with such elements, and this trend shows no signs of abating. This trend is not limited to the United Kingdom, and the use of botanical preparations in antiaging products has increased from 63.8% in 2011 to 73.8% in 2018 [1, 6]. Despite the undeniable appeal of medicinal plants or herbal medicines, safety concerns necessitate responsible engagement. Potential hazards are numerous, including contamination, misidentification, and inadequate testing for toxicity and adverse effects. These shortcomings pose significant threats, particularly in the realm of herbal beauty products [7, 8].

Callus culture, a plant tissue and cell culture technique, has emerged as a potential remedy, offering a controlled and sustainable platform for overcoming these challenges. This innovative technique promotes the growth of undifferentiated plant cells under controlled conditions, presenting a plethora of advantages [9]. Callus culture enables mass production of bioactive compounds in a sterile environment, mitigating the variability and contamination risks inherent in sourcing these compounds from whole plants. This translates to a more consistent and reliable supply of botanical ingredients, which is beneficial for the health and cosmetic industries [10]. Moreover, callus culture empowers the targeted production of specific bioactive compounds, bypassing the need for whole‐plant harvesting and thereby alleviating concerns about overexploitation and environmental impact. This sustainable approach aligns with the growing desire for ethically and environmentally responsible sourcing, a cornerstone of responsible product development [10, 11]. In addition to addressing existing challenges, callus culture presents a fascinating platform for research and development. It facilitates the study and production of bioactive compounds with diverse health and cosmetic applications. Scientists can isolate specific compounds with desired properties, paving the way for the creation of novel formulations and products [12]. Because terms such as “anti‐aging,” “cosmeceutical,” and “geroprotective” are used inconsistently across the literature, we define them here as used in this review. By anti‐aging we refer to interventions that measurably preserve or restore the structure and function of aged or photoaged skin. A cosmeceutical is a topically applied product positioned between a cosmetic and a drug, claiming a bioactive benefit beyond simple adornment, and geroprotective signifies an effect that slows or counteracts cellular and tissue‐level hallmarks of aging, such as oxidative damage, aging, and extracellular matrix degradation. To keep the remainder of this review focused, we organize the mechanistic discussion around a defined set of endpoints most relevant to cutaneous aging: (i) antioxidant and reactive‐oxygen‐species (ROS) modulation, (ii) collagen synthesis and matrix metalloproteinase (MMP) inhibition, (iii) barrier and hydration markers (e.g., aquaporin 3, transepidermal water loss), (iv) inflammatory mediators (COX‐2, iNOS, IL‐1*β*), (v) pigmentation and tyrosinase activity, and (vi) clinical outcomes (wrinkle scores, elasticity, dermal density). Throughout, we distinguish whether a given finding is based on acellular biochemical assays, cell‐based models, or human studies, since these carry different weights of evidence.

## 2 Callus Culture and Skin Care

The concept of totipotentiality, which is now known as totipotency, was introduced in 1902. All plant cells have the potential to give rise to a complete plant. This idea laid the foundation for the development of tissue and cell culture methods, leading to significant advancements in the fields of biology and medicine. This early vision in research and pioneering work in the systematic culture of single cells in vitro profoundly affected the understanding of plant cell behavior and the realization of tissue culture techniques [11, 13].

**FIGURE 1:**
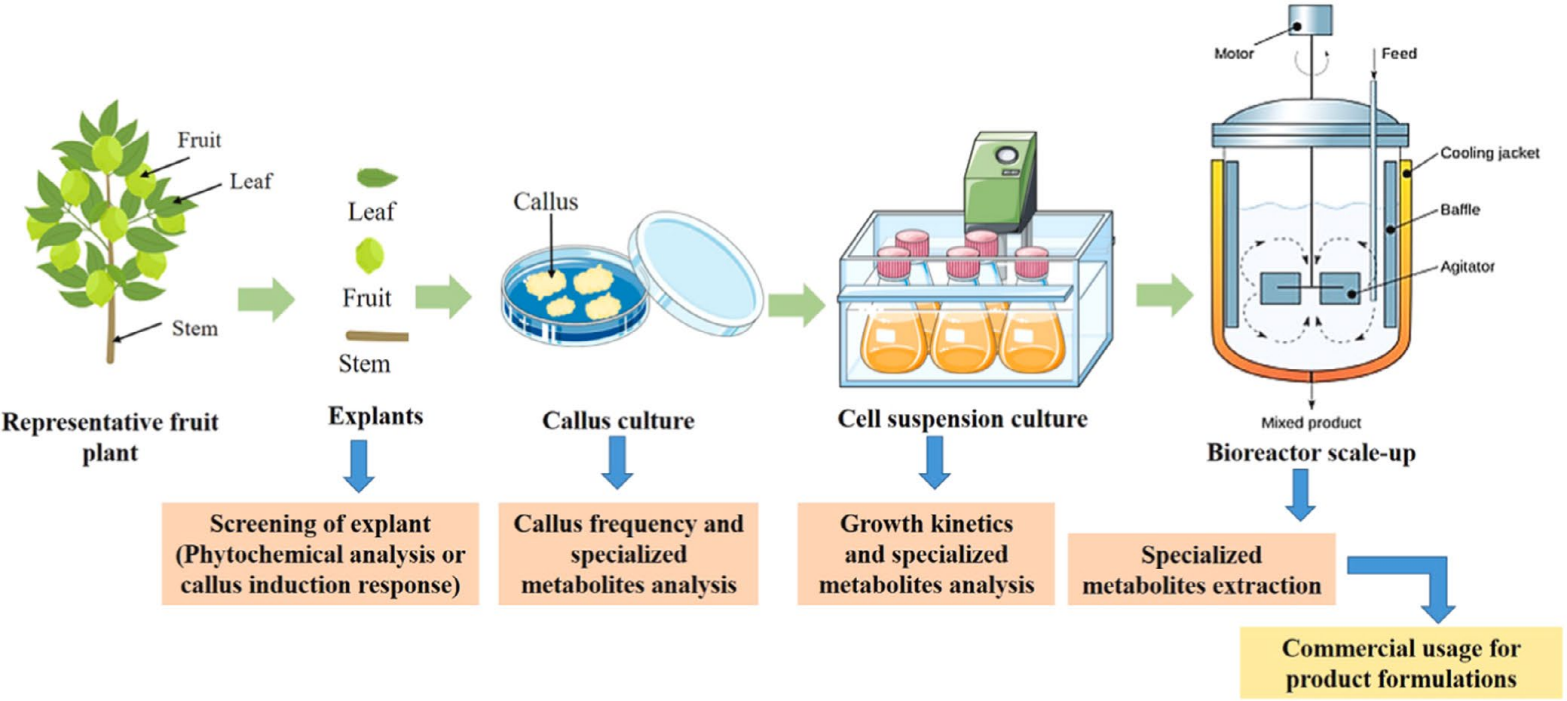
From callus to finished products [21].

Callus culture is a crucial biotechnological technique that involves the in vitro culture of dedifferentiated plant cells, or calli, on media containing elevated levels of auxin and/or cytokinin. These plant hormones play a synergistic role in regulating various aspects of plant growth and development, including cell division, elongation, and morphogenesis [14, 15]. By manipulating the auxin–cytokinin ratio in the culture medium, researchers can induce calli to produce a wide range of secondary metabolites, which are compounds with valuable medicinal, dietary, and cosmetic properties [16, 17]. The use of plant cell cultures for the production of secondary metabolites offers several advantages over traditional agricultural methods. This approach provides a consistent and controlled manufacturing process, independent of seasonal variations, and has a lower environmental impact, reducing water usage, the carbon footprint, and the need for pesticides and herbicides [11, 18]. However, commercial production processes using plant cell cultures for secondary metabolites, particularly in the pharmaceutical industry, are limited due to somaclonal variations and low metabolite yields. In contrast, the cosmetics industry, driven by consumer demand for effective, safe, natural, and sustainable products, has shown high interest in plant cell culture extracts with specific activities for skincare, makeup, and hair care. These extracts can be produced under controlled conditions, even from rare or endangered plant species, and can be used at minimal concentrations in final cosmetic formulations. The low product yield is less critical in cosmetic applications, and the use of plant cell culture extracts has contributed to a renaissance in plant cell culture technology, leading to the production of many cosmetic products over the past decade [19, 20]. The production of therapeutic compounds via callus culture typically involves two steps: the induction of rapid callus growth and the stimulation of secondary metabolite production. Figure 1 shows a step‐by‐step overview of the processes that lead to the transformation of the callus culture into the final product. The optimal conditions for each step vary depending on the plant species and the desired metabolite. However, in general, calli require specific levels of light, temperature, humidity, and nutrients for optimal growth and development. These elements constitute the second most common impact factors, known as biotic factors (pectin, pectic acid, chitin, chitosan, and glucans) and abiotic factors (extremes of temperature, ultraviolet light, osmotic pressure, antibiotics, fungicides, and salts) [15, 22].

To clarify how culture conditions translate into skin‐relevant outcomes (Table 1), maps the principal culture levers used in callus systems to the metabolite classes they tend to enrich and to the skin‐relevant actions those metabolites are reported to exert. This illustrates that callus culture is not merely a sustainability strategy but a tunable biosynthetic platform.

**TABLE 1:** Mapping of callus culture levers to enriched metabolite classes and plausible skin‐relevant actions.

Culture lever	Typically enriched metabolite classes	Plausible skin‐relevant action(s)
High auxin/cytokinin ratio	Drives callus proliferation/biomass; baseline phenolic and flavonoid pools	Antioxidant capacity; substrate supply for downstream actives
Biotic elicitors (chitosan, chitin, glucans, pectin)	Phenylpropanoids, flavonoids, phytoalexins (defense metabolites)	ROS scavenging; anti‐inflammatory signaling (NF‐κB/AP‐1 attenuation)
Abiotic elicitors: salicylic acid, jasmonic acid	Anthraquinones, total flavonoids (often dose‐dependent; may reduce biomass)	UV absorption (reported SPF gains); photoprotection
Light quality/UV exposure (e.g., LED wavelengths)	Phenolics, flavonoids, (neo)lignans; extracellular vesicle yield	Reduced melanin/ROS; anti‐pigmentation; antioxidant defense
Osmotic/salt or temperature stress; precursor feeding (e.g., phenylalanine)	Phenolic acids (rosmarinic, caffeic, ferulic), terpenoids	Antioxidant and photoprotective capacity; collagenase/elastase inhibition

*Note:* Associations are indicative and species‐dependent; effects on biomass and metabolite yield often trade off, and skin‐relevant actions are inferred from the listed metabolite classes rather than demonstrated for every lever–species combination.

## 3 Skincare and Anti‐Aging Products

In the contemporary era, the advancement of cosmeceuticals has progressed toward integrating efficacious constituents aimed at assisting consumers in preserving youthful and radiant visage, surpassing conventional practices of cleansing and moisturizing [23]. The domain of antiaging is among the most vibrant in the spectrum of cosmetics in terms of both the rapidity and comprehensiveness of its advancements. There is a notable and escalating consumer demand for fresh and enhanced methodologies and ingredients to counteract, postpone, and prevent manifestations of skin aging [24]. Among emerging delivery systems, lipid‐based nanoparticles such as SLNs and NLCs are increasingly recognized for their ability to improve the stability and dermal absorption of cosmetic actives, offering valuable potential for incorporation into anti‐aging skincare formulations [11]. In particular, most favor botanical and plant‐derived components [10].

The visible signs of aged skin encompass a diverse array of characteristics, reflecting the intricate interplay of various physiological processes. These features include wrinkles, dryness, laxity, irregular pigmentation, and a rough skin texture [25, 26]. However, the aging process extends beyond the surface, as aged skin also exhibits functional impairments in its barrier, immune response, wound healing, and sensory perception capabilities [27, 28]. The appearance of aged skin can vary significantly, with a distinct contrast between intrinsically aged skin and photoaged skin. Whereas intrinsically aged skin typically presents as thin, finely wrinkled, and dry, premature photoaged skin often displays a thickened epidermis, mottled pigmentation, telangiectasias (dilated blood vessels), and coarse wrinkles [26, 29, 30].

**FIGURE 2:**
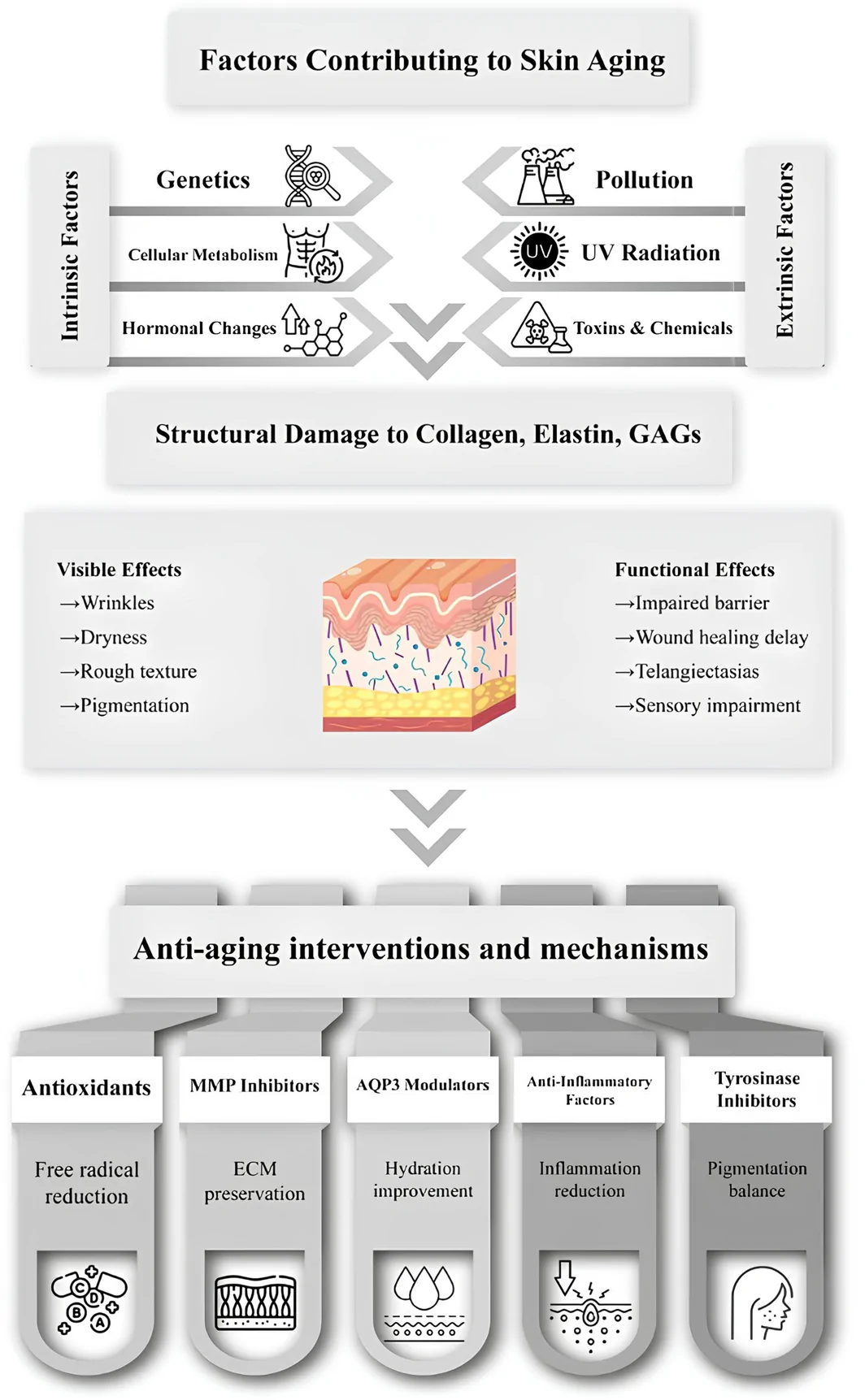
A comprehensive look at factors affecting skin aging and possible intervention.

The underlying mechanisms driving skin aging are multifaceted and complex and involve a combination of intrinsic and extrinsic factors. Intrinsic factors, such as genetics, cellular metabolism, and hormonal and metabolic processes, are intertwined with extrinsic factors, including chronic exposure to light, pollution, radiation, toxins, and chemicals, to orchestrate the aging process [31, 32]. At the core of this intricate tapestry are the primary structural components of the dermis: collagen, elastin, and glycosaminoglycans (GAGs). These elements have been the focal point of extensive antiaging research and the development of aesthetic‐antiaging strategies, ranging from antiwrinkle creams to various filling agents [33, 34, 35]. Two main groups of agents have emerged as potential antiaging interventions: antioxidants and cell regulators [26]. Antioxidants, such as vitamins, polyphenols, and flavonoids, aim to reduce collagen degradation by mitigating the concentration of free radicals in tissues. Conversely, cell regulators, including retinol, peptides, and growth factors, exert direct effects on collagen metabolism and influence collagen production [26, 36]. A deep visualization of the skin aging process, the factors involved, and important groups of antiaging agents is shown in Figure 2.

## 4 Molecular Mechanisms Underlying Skin Aging

In the realm of dermatological research, a diverse array of analytical methodologies is employed to evaluate the efficacy of potential anti‐aging compounds and formulations. The preponderance of these investigative procedures focuses on assessing the antioxidative capabilities of these substances [32, 37]. Topically applied antioxidants mitigate cutaneous senescence through multiple mechanisms, including the attenuation of oxidative stress and the preservation of mitochondrial functionality. Furthermore, these compounds exert their geroprotective effects via the modulation of inflammatory cascades, DNA safeguarding, apoptosis inhibition, autophagy promotion, and the regulation of various signaling pathways, such as the MAPK, TGF‐*β*, and hedgehog pathways [38, 39].

To quantify the antioxidant potency of these substances, particularly those derived from botanical sources, researchers utilize an extensive battery of assays. These include but are not limited to 2,2‐diphenyl‐1‐picrylhydrazyl (DPPH) radical scavenging assays, hydrogen peroxide (H_2_O_2_) assays, total phenolic, polyphenolic, and flavonoid content determination, oxygen radical absorbance capacity (ORAC) assays, 2,2′‐azino‐bis (3‐ethylbenzothiazoline‐6‐sulphonic acid) (ABTS) assays, cupric ion reducing antioxidant capacity (CUPRAC) assays, metal chelation activity assays, 2,2′‐bipyridyl‐6,6′‐bis (2‐benzothiazolyl) pyridine (BTS) radical scavenging assays, protein glycation assays, potassium ferricyanide reducing power (PFRAP) assays, lipid peroxidation inhibition (LPO) assays, and superoxide dismutase activity (SOD) assays.

Notably, these aforementioned assays are conducted in acellular environments and utilize extracts and isolated bioactive compounds. To further elucidate the biological effects of these substances, additional investigations have been performed on living cells or tissues. These may encompass the quantification of intracellular reactive oxygen species (ROS) levels via flow cytometry or alternative laboratory techniques and oxidative stress challenge assays. This comprehensive approach for evaluating antioxidant efficacy provides a multifaceted understanding of the potential geroprotective properties of various compounds and formulations in the context of cutaneous aging. It is important to caution, however, that acellular antioxidant assays such as DPPH, ABTS, ORAC, and CUPRAC measure intrinsic chemical radical‐scavenging capacity only; they do not account for skin penetration, bioavailability, intracellular partitioning, or metabolic transformation, and therefore should not be interpreted as direct evidence of clinical anti‐aging benefit. For efficacy claims, cell‐based and tissue‐based endpoints carry greater weight, including reduction of intracellular ROS in keratinocytes and fibroblasts, suppression of MMP‐1 (and related MMPs), induction of type I/III collagen and procollagen, attenuation of inflammatory cytokines, and, ultimately, validated human outcomes. Throughout the remainder of this review we accordingly prioritize cell‐based and clinical endpoints over acellular assays when assessing the strength of an anti‐aging claim.

Skin wrinkles, as one of the main signs of skin aging, have attracted the attention of researchers and require finding the root of the phenomenon and investigating the factors involved and ways to prevent these causes [40, 41]. The attenuation of cutaneous senescence and dermal matrix degradation is intricately linked to the modulation of matrix metalloproteinases (MMPs), particularly MMP‐1, MMP‐2, and MMP‐9. These zinc‐dependent endopeptidases play pivotal roles in extracellular matrix (ECM) remodeling and collagen catabolism [42, 43]. MMP‐1, also known as interstitial collagenase, is a key mediator of dermal collagen degradation, and its upregulation is strongly associated with photoaging and rhytid formation [44]. MMP‐2 (gelatinase A) plays a more nuanced role, with elevated levels paradoxically correlated with longevity in certain populations, suggesting a complex interplay between its activity and dermal homeostasis [45, 46]. While less extensively studied in the context of cutaneous aging, MMP‐9 (gelatinase B) contributes to ECM degradation and is often concomitantly upregulated with other MMPs in response to ultraviolet radiation. Inhibition of these metalloproteinases, particularly MMP‐1, has emerged as a promising strategy for combating dermal atrophy and maintaining skin elasticity [42, 45, 46]. Novel approaches, such as the application of peptide nucleic acid derivatives targeting MMP‐1 gene silencing, have demonstrated efficacy in augmenting collagen synthesis and ameliorating clinical signs of photodamage [44]. Furthermore, the suppression of MMP‐2 and MMP‐9 expression through various pharmacological interventions, including the use of phytochemicals and synthetic inhibitors, has shown potential in mitigating the deleterious effects of UV‐induced dermal matrix degradation and preserving cutaneous integrity [31, 47, 48].

The cutaneous hydration state is intricately linked to the expression and functionality of aquaporin 3 (AQP3), a transmembrane protein that facilitates the transcellular flux of water and glycerol [49, 50]. The pivotal role of AQP3 in epidermal homeostasis is evidenced by its ability to modulate stratum corneum hydration, keratinocyte proliferation, and dermal elasticity [51, 52]. The upregulation of AQP3 gene expression is correlated with increased epidermal water retention and reduced transepidermal water loss, thereby increasing the moisture content and barrier function of the skin [49, 50]. Various exogenous compounds, including phytochemicals and cannabinoids, have demonstrated efficacy in stimulating AQP3 expression, consequently ameliorating cutaneous hydration [53, 54]. The mechanistic underpinnings of this phenomenon involve the facilitation of osmotically driven water transport and glycerol permeation across cellular membranes, culminating in improved dermal turgor and plasticity [50]. Thus, targeting AQP3 represents a promising strategy for enhancing skin moisturization and maintaining optimal epidermal homeostasis. Moreover, the role of AQP3 extends to anti‐aging effects by promoting keratinocyte proliferation and migration, which are vital for skin repair and regeneration [55].

Another significant determinant of the senescence of cutaneous tissue is chronic low‐grade inflammation, a phenomenon exacerbated by both intrinsic and extrinsic factors [56, 57]. This inflammaging process is characterized by the upregulation of proinflammatory mediators, including cyclooxygenase‐2 (COX‐2), inducible nitric oxide synthase (iNOS), tumor necrosis factor‐alpha (TNF‐*α*), interleukin‐1 alpha (IL‐1*α*), interleukin‐1 beta (IL‐1*β*), and prostaglandin E2 (PGE2). These molecular effectors orchestrate a complex cascade of events that culminate in dermal matrix degradation, epidermal atrophy, and compromised barrier function [56, 58]. COX‐2, a key enzyme in prostanoid biosynthesis, catalyzes the production of PGE2, which further amplifies the inflammatory response and contributes to collagen degradation [59]. Concomitantly, iNOS generates nitric oxide, potentiating oxidative stress and cellular senescence. The proinflammatory cytokines TNF‐*α*, IL‐1*α*, and IL‐1*β* act synergistically to activate the transcription factors nuclear factor kappa B (NF‐κB) and activator protein 1 (AP‐1), thereby upregulating matrix metalloproteinases and further compromising dermal integrity [60, 61, 62]. Targeting these inflammatory mediators through pharmacological interventions or nutraceutical compounds represents a promising strategy for mitigating the deleterious effects of cutaneous aging, potentially restoring dermal homeostasis and preserving skin elasticity and functionality [58, 63, 64, 65].

Cutaneous senescence is inextricably linked to the modulation of melanogenesis and tyrosinase activity, a copper‐dependent oxidase that catalyzes the rate‐limiting step in melanin biosynthesis [66]. Hyperactivation of tyrosinase precipitates hyperpigmentary disorders, notably senile lentigines, which are quintessential manifestations of photoaged skin [67]. Consequently, tyrosinase inhibitors have emerged as pivotal agents for attenuating these effects by suppressing melanin overproduction and promoting cutaneous chromatic homeostasis [66, 68]. The antiaging efficacy of these compounds is further potentiated by their capacity to inhibit matrix‐degrading enzymes such as elastase, collagenase, and hyaluronidase, which are involved in dermal extracellular matrix degradation, culminating in diminished skin elasticity and rhytid formation. Multifunctional agents that exhibit both tyrosinase inhibitory properties and matrix metalloproteinase suppression offer a comprehensive approach to combating the multifaceted aspects of skin aging, preserving dermal architecture and function while mitigating hyperpigmentation and maintaining a youthful cutaneous phenotype [67, 68, 69, 70].

## 5 Plant Callus Culture and UV Filters for Photoaging Protection

**FIGURE 3:**
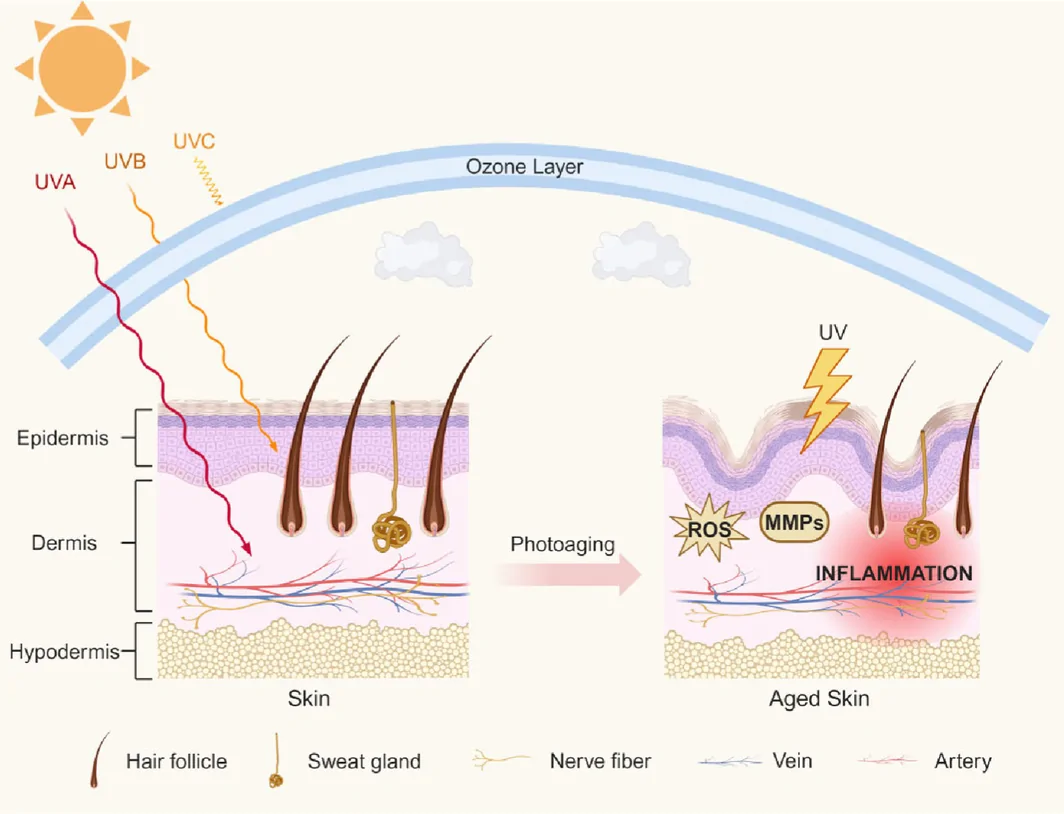
UV light causing inflammation and photoaging [73]. Used under Creative Commons CC‐BY license. No changes were made.

**FIGURE 4:**
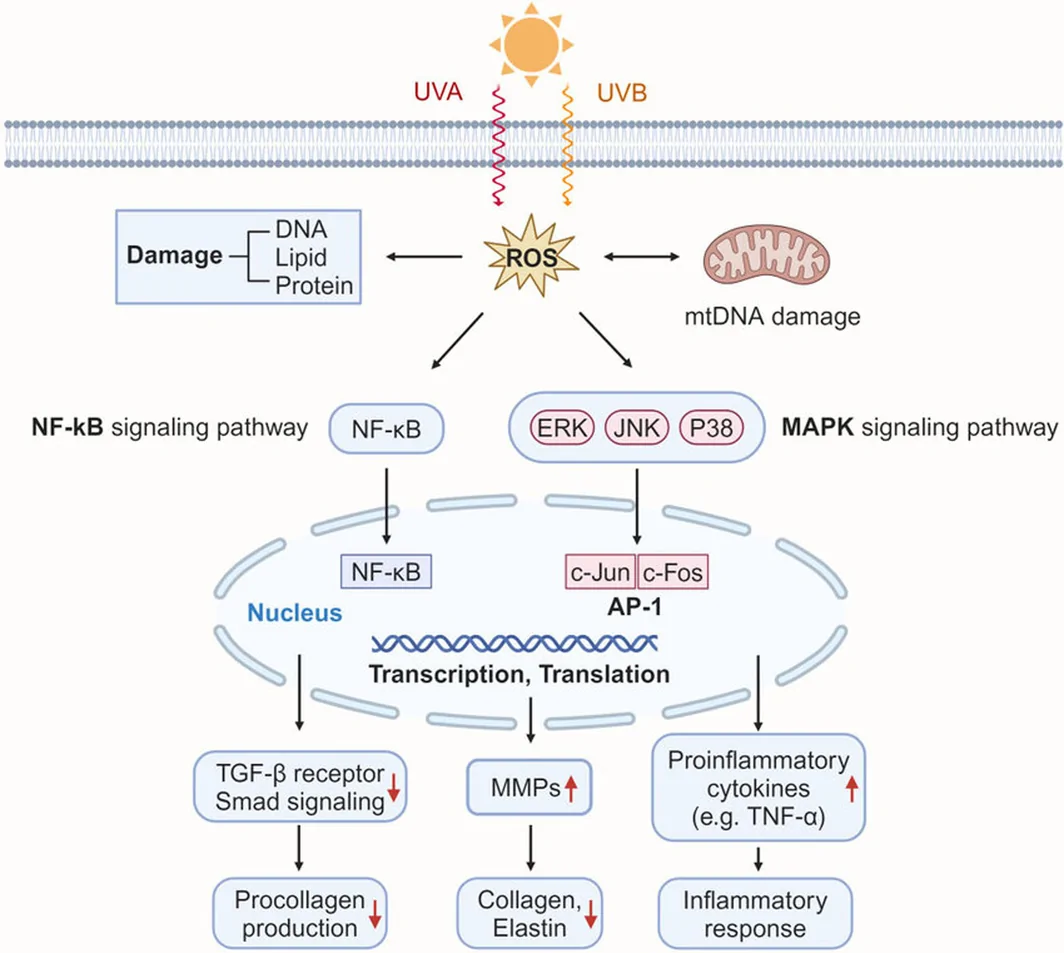
A detailed demonstration of pathways that originate from UVA and UVB exposure [73]. Used under Creative Commons CC‐BY license. No changes were made.

UV rays can trigger the production of free radicals, harming mitochondrial enzymes and plasma membranes as well as lowering the skin's antioxidant levels [71]. Moreover, UVB of sunlight interacts with cellular chromophores in the skin's upper epidermis, leading to DNA damage, which is associated with photoaging, photo immunosuppression, photocarcinogenesis, and skin burns [72]. Figure 3 demonstrates UV light inducing ROS, causing skin inflammation and photoaging. Figure 4 shows the mechanism in full detail.

Before discussing specific examples, it's important to discuss how sun protection factor (SPF) is measured, because the values reported for plant callus extracts vary widely in their methodological basis. The SPF values cited below are, in most cases, in vitro spectrophotometric estimates calculated from the absorbance of an extract or fraction (e.g., the Mansur equation), rather than standardized in vivo SPF determinations or formulation‐based testing performed under ISO 24444 (in vivo) or ISO 24443 (in vitro UVA) protocols. Spectrophotometric SPF of a raw extract does not account for the final formulation matrix, film thickness, photostability, substantivity, or actual application conditions, and therefore tends to overestimate real‐world protection; such figures should be regarded as screening indicators only. Equally important, photoprotection is not exclusively a function of UV absorption: a meaningful photoprotective ingredient should also reduce UV‐induced oxidative stress, inflammation (e.g., COX‐2/PGE2), and DNA damage in relevant skin models. We therefore present the SPF figures below as preliminary indicators of UV‐absorbing potential, to be confirmed by standardized formulation‐based testing and complemented by cell‐based readouts of UV‐induced damage.

In response to this threat, plant‐derived bioactive compounds have evolved as a natural defense mechanism against UV radiation. Given that plants are sessile organisms, they are continuously exposed to sunlight and have developed complex biochemical pathways to synthesize compounds capable of absorbing or neutralizing harmful UV rays [74]. Notably, compounds such as flavonoids, phenolics, and carotenoids not only provide protection for plants but also exhibit substantial UV‐absorbing and antioxidant properties beneficial for human skin. This is due to the nature of their chemical composition, which enables them to interact with free radicals with several mechanisms; the most common mechanism is donating hydrogen atoms to the free radicals. These bioactive compounds act as natural UV filters by absorbing UV‐A (320–400 nm) and UV‐B (280–320 nm) radiation, thus mitigating DNA damage, oxidative stress, and inflammation induced by free radicals. Furthermore, their antioxidant properties enhance skin protection by scavenging reactive oxygen species (ROS) generated from UV exposure [75, 76]. For example, anthraquinones, which are naturally occurring phenolic compounds in plants of the Fabaceae family, particularly *Cassia tora* , are esteemed for their chromatic, therapeutic, and garnered demand across diverse sectors such as cosmetics, food, dyes, and medicine [77]. Key anthraquinones from *Cassia tora* include emodin, rhein, chrysophanol, obtusin, obtusifolin, physcion, and cassiaside, which have been isolated from various plant parts and demonstrated a range of biological activities, including larvicidal, anti‐tyrosinase, anti‐diabetic, antifungal, and antimicrobial effects [78, 79]. A recent manuscript reported that the cultivation of *Cassia tora* callus, combined with salicylic acid as an elicitor, could result in a 10–50 fold increase in anthraquinone concentration relative to that found in natural seeds, achieving a maximum SPF of 38. In this study, various fractions of anthraquinone compounds from the acidified 75% methanol extract of the callus of this plant were prepared and the SPF of these fractions was investigated [78].

A study investigating two varieties of *Butea monosperma* explored the use of an elicitor, jasmonic acid, to enhance sun protection factor (SPF) values, but these efforts were not completely successful. In one variety, treatment with jasmonic acid, an elicitor, had little effect, while in the other variety, it significantly increased the total flavonoid content (TFC) in a dose‐dependent manner. However, the application of jasmonic acid was generally associated with a reduction in biomass. The maximum SPF values recorded from the callus extracts of the two varieties were 19 and 14.6, respectively, highlighting their potential for sun protection. Additionally, a positive correlation was noted between the TFC in the callus of one variety and its corresponding SPF level. High‐performance liquid chromatography‐mass spectrometry (HPLC–MS) analysis identified several bioactive flavonoids in the extracts, including formononetin, butin, and isoliquiritigenin, which are known for their skin‐protective properties. Flavonoids, which are often responsible for the pigmentation in plants, possess aromatic and cyclic structures that enable them to absorb ultraviolet (UV) light within the 240–285 nm and 300–550 nm wavelength ranges, contributing to their role in UV protection [80].

A recent study on the UV‐protective properties of plants focused on the plant *Ocimum basilicum* . Analysis of the phenolic profile of *Ocimum basilicum* (sweet basil) reveals a complex composition, primarily featuring rosmarinic, vanillic, lithospermic, hydroxybenzoic, coumarinic, syringic, ferulic, protocatechuic, and caffeic acids in this study and controlled laboratory conditions, exogenous application of phenylalanine and salicylic acid demonstrates a stimulatory effect on the biosynthesis of these phenolic compounds within in vitro sweet basil callus cultures, surpassing levels observed in untreated controls. Further investigation into the photoprotective properties of ethanolic extracts derived from phenylalanine‐elicited callus cultures (two‐week exposure) indicates a substantial sun protection factor (SPF), reaching a maximum value of 36.5. This elevated SPF value suggests a significant potential for the incorporation of these elicited callus extracts into topical formulations designed for photoprotection, providing a biogenic alternative for use as a component in sunscreen products [81].

While the aforementioned studies provide valuable insights into the photoprotective potential of different callus extracts, specifically concerning their UV‐filtering capacity, it is important to acknowledge that this represents only one facet of a complex mechanism. A comprehensive understanding of the role of plant callus extracts in preventing photoaging necessitates further investigation into mechanisms beyond direct UV absorption. For instance, research exploring the broader biological activity of such extracts is exemplified by a study on *Mesembryanthemum crystallinum* . Investigations into the effects of *M. crystallinum* extracts on gene expression in human dermal fibroblasts have revealed positive modulation of genes associated with antioxidant defense and collagen synthesis [82]. This exemplifies the need for expanded research paradigms that delve into the multifaceted effects of plant callus extracts, encompassing not only UV filtration but also their influence on cellular processes crucial for mitigating photoaging, such as the modulation of gene expression related to cellular repair, structural integrity, and oxidative stress response.

## 6 Callus Culture and Antiaging Effects

**FIGURE 5:**
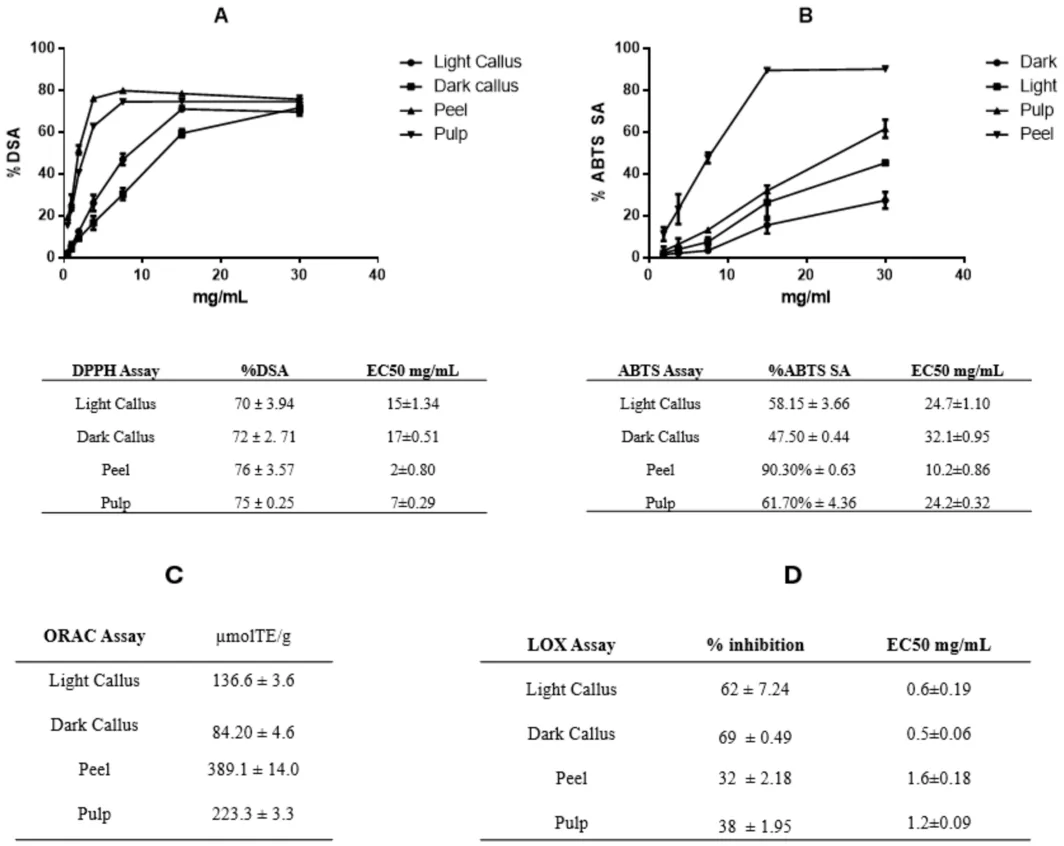
DPPH (A), ABTS (B), ORAC (C) tests, and lipoxygenase inhibition activity (D). Showing better inhibition as the extract concentration increases [83]. Used under Creative Commons CC‐BY license.

**FIGURE 6:**
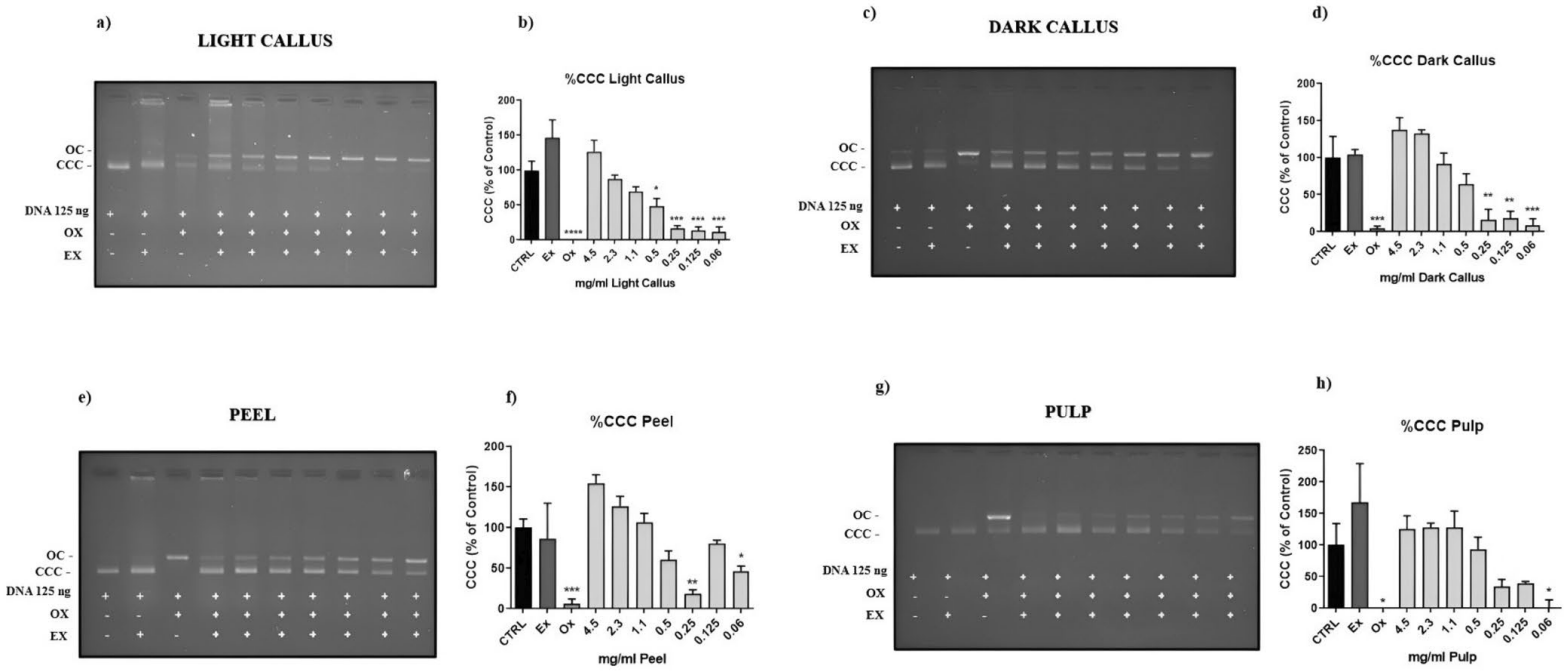
Protective effect of Annurca apple extracts against strand breaks using the DNA nicking assay [83]. Used under Creative Commons CC‐BY license.

The utilization of callus culture for antiaging applications is predicated on the remarkable ability of these undifferentiated cells to synthesize and accumulate secondary metabolites, often at higher concentrations than those found in the parent plant. These bioactive compounds, including polyphenols, flavonoids, and terpenoids, exhibit potent antioxidant, anti‐inflammatory, and cytoprotective properties, which are crucial in combating the multifaceted processes of cellular senescence and organismal aging [83, 84, 85]. Figures 5 and 6 portray the discussed properties and effects, respectively.

**FIGURE 7:**
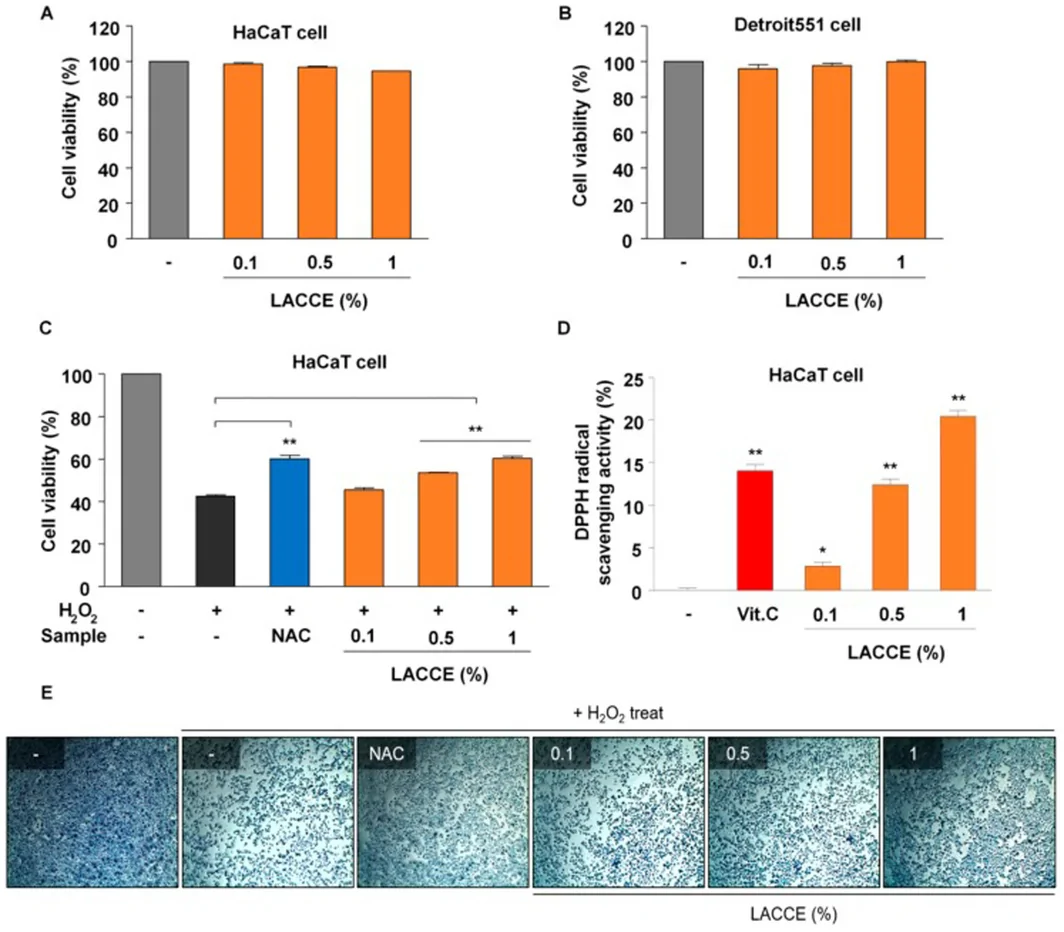
In vitro assessment of LACCE as an anti‐aging agent, including cytotoxicity and anti‐oxidant activity [86]. Used under Creative Commons CC‐BY license.

**FIGURE 8:**
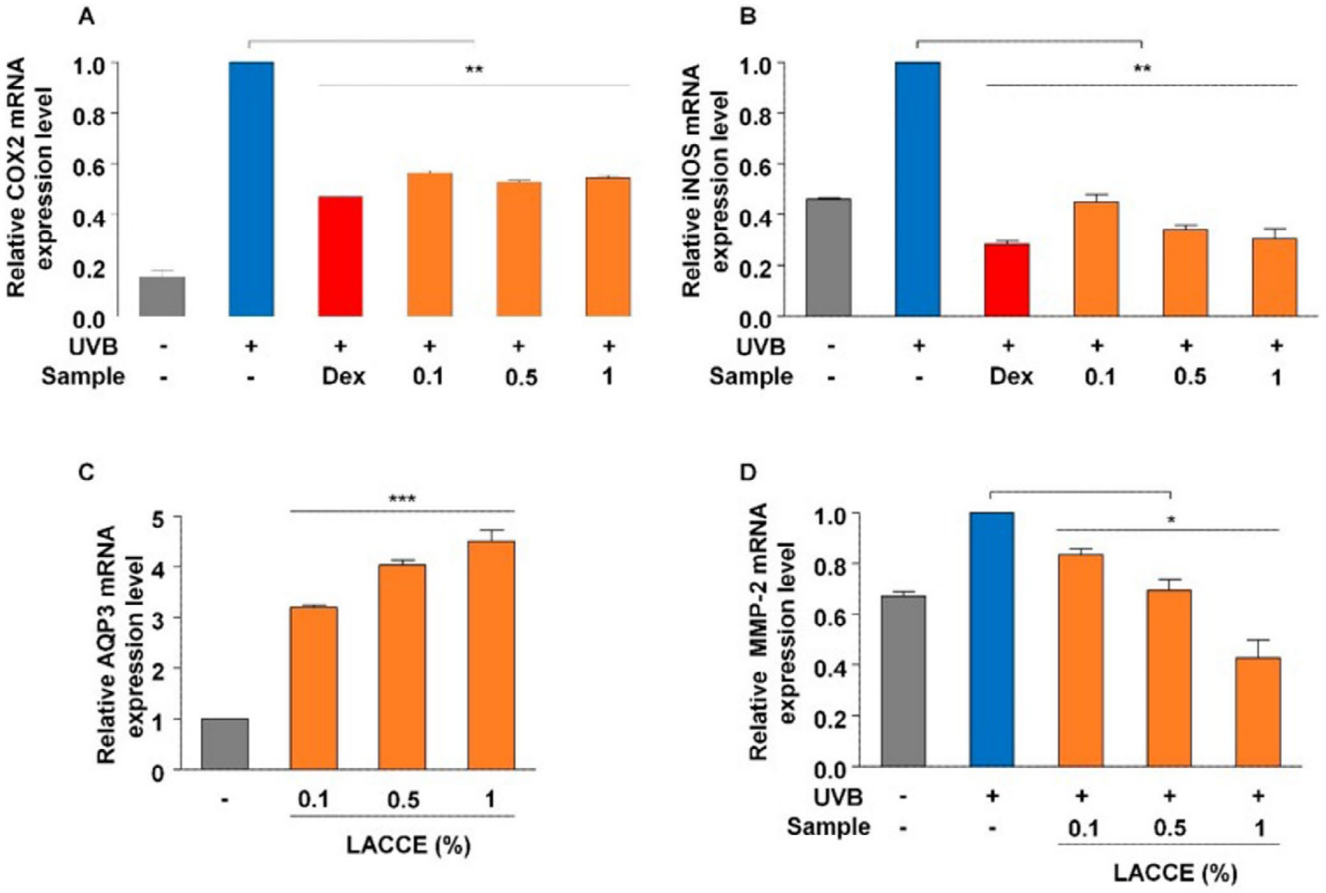
In vitro assessment of LACCE as anti‐inflammatory, moisturizing, and anti‐wrinkle agents by real‐time RT‐PCR [86]. Used under Creative Commons CC‐BY license.

One exemplary case is the callus culture of Leontopodium alpinum, colloquially known as Edelweiss. Extracts derived from *L. alpinum* callus cultures have demonstrated significant antiaging effects through various mechanisms. Transcriptome profiling of human keratinocytes treated with *L. alpinum* callus culture extract (LACCE) revealed the upregulation of genes involved in keratinization, cornification, and skin barrier formation. Concomitantly, LACCE downregulated stress‐responsive genes associated with oxidation, wounding, and hypoxia, suggesting a comprehensive protective effect on skin cells. As shown in Figure 7, in vitro assays have corroborated the potent antioxidant activity of LACCE, particularly in response to UVB‐induced oxidative stress. Furthermore, clinical studies have shown that topical application of LACCE improves various parameters of skin aging, including periorbital wrinkles, skin elasticity, and dermal density (Figure 8) [86, 87].

Another noteworthy example is the callus culture of *Centella asiatica* , a medicinal plant renowned for its diverse therapeutic properties. The ethanolic extract of *C. asiatica* callus culture has been found to contain distinctive antioxidant compounds, as elucidated by HPTLC‐DPPH and HPLC analyses. In human dermal fibroblasts, this extract demonstrated cytoprotective effects against hydrogen peroxide‐induced oxidative stress, primarily through the upregulation of cellular antioxidant enzymes. Moreover, the extract inhibited the induction of matrix metalloproteinase‐9, a key enzyme involved in extracellular matrix degradation and skin aging (Table 2). Papers related to the applications of calluses in the manufacture of antiaging products have been published according to the findings and analyses presented in Table 3.

**TABLE 2:** Proposed minimal standard panel of assays and reporting items for future callus‐culture anti‐aging studies.

Domain	Recommended assays/reporting items	Minimum to report
Culture & material provenance	Species and tissue source; explant; basal medium and hormone (auxin/cytokinin) regime; elicitor and dose; light/temperature; passage number	Full culture conditions; extract solvent and yield
Metabolite profiling	HPLC/LC–MS or HPTLC fingerprint; quantification of ≥ 1 marker compound; total phenolic/flavonoid content as supportive data	Marker concentration with units; chromatogram
Stability	Marker retention over time/temperature/light; pH sensitivity; stability in the final formulation matrix	% marker remaining vs. time/condition
Cell‐based efficacy	Viability/cytotoxicity; intracellular ROS in keratinocytes/fibroblasts (±UV challenge); MMP‐1 (±MMP‐2/9); type I procollagen/collagen; key inflammatory markers (e.g., IL‐1*β*, COX‐2); tyrosinase/melanin where pigmentation is claimed	Cell line, dose range, positive control, replicates, statistics
Penetration/delivery	Franz diffusion cell and/or ex vivo skin permeation; tape‐stripping for stratum corneum deposition; carrier characterization if encapsulated (size, zeta, encapsulation efficiency)	Flux/recovery of marker; membrane/skin model used
Safety	Irritation and phototoxicity (e.g., reconstructed‐epidermis or 3 T3 NRU); sensitization assessment; microbial/endotoxin and preservative‐efficacy data for the final product	Method, concentration tested, outcome
Human outcomes	Controlled clinical study with instrumented endpoints: wrinkle scores, cutometer elasticity, hydration/TEWL, and pigmentation (e.g., melanin index), with adequate sample size and duration	*n*, duration, design, blinding, statistics

**TABLE 3:** Applications of calluses in antiaging products according to the findings and analyses.

Plant species	Abstract	Assays in brief	Ref.
Leontopodium alpinum (Edelweiss)	Leontopodium Alpinum callus culture extract (LACCE) showed strong antioxidant activity in response to UVB treatment. LACCE improved anti‐periorbital wrinkles, skin elasticity, and dermal density. LACCE up‐regulated genes for skin barrier formation and down‐regulated stress‐responsive genes. LACCE is a promising agent for anti‐aging cosmetics.	Cytotoxicity by MTT assay test Antioxidant Activity: DPPH Radical Scavenging Assay. Hydrogen Peroxide (H_2_O_2_) Assay on HaCaT cells. Real‐time RT‐PCR: ‐Anti‐wrinkles, moisturizing, and anti‐inflammation effects respectively associated with expression of matrix metalloproteinase‐2 (MMP‐2), Aquaporin 3 (AQP3) and COX2 and iNOS, both for anti‐inflammatory effects. Clinical test: ‐On a total of 21 female volunteers in different ages over 4 weeks for four different factors: facial lifting and improving periorbital wrinkles, skin elasticity, dermal density, and skin thickness.	[86]
Leontopodium alpinum L. (Edelweiss)	Magenta‐LED increased extracellular vesicles production by 2.6‐fold compared to darkness. Magenta‐LED exposed callus reduced ROS production and melanin content effectively. M‐LED enhanced callus growth and secondary metabolite production significantly. Extracellular vesicles in Magenta‐LED exposed callus showed higher skin protein expression and reduced melanin.	Cell Viability by HaCaT cells (human keratinocytes), Detroit cells (human fibroblasts), and B16F10 cells (mouse‐derived melanoma cells). Analysis of Total Phenolic Compounds (TPC) and Total Flavonoid Compounds (TFC). Measurement of Intracellular ROS Levels H2DCF‐DA experiment was conducted using HaCaT cells. Measurement of Extracellular Melanin Production using B16F10 melanoma cells. Western Blot Analysis	[88]
Leontopodium alpinum L. (Edelweiss)	Ten active components, including chlorogenic acid (A), isoquercitrin (B), isochlorogenic acid A (C), cynaroside (D), syringin (E), isochlorogenic acid (F), cynarin (G), rutin (H), leontopodic acid A (I), and leontopodic acid B (J)screened and identified for blue light‐damageprotecting activities and mechanisms. LACCE components reduced ROS, Ca 2+ influx, and OPN3 expression. Leontopodic acid A boosted COI‐1, hindered MMP‐1, and reduced OPN3. LACCE active compounds have anti‐blue light damage effects on fibroblast models.	Cytotoxicity assessment by cell counting kit‐8. Measurement of COL‐I, MMP‐1, and OPN3 Levels Using ELISA and Western blot on Human Foreskin Fibroblasts (HFF). Western blotting and ELISA for mechanism exploration. ROS secretion and Ca 2+ influx measurement by flow cytometry	[87]
*Oryza sativa* varieties (Hommali 105 (nonpigmented), Munpu (red rice), and Niawdum (black rice))	Munpu callus had highest anti‐oxidant, keratinocyte proliferation, and anti‐aging activities. Calli rich in phenolic contents, amino acids, and bioactive compounds.	Cytotoxicity by MTT assay test Antioxidant activity: Total Phenolic Content (TPC) Determination Potassium Ferricyanide Reducing Power Assay (PFRAP) DPPH Radical Scavenging Assay (DPPH) Lipid Peroxidation Inhibition (LPO) Superoxide Dismutase Activity (SOD) Promoting Keratinocyte Proliferation by the modified MTT method on the NHEK cells Anti‐Collagenase Activity by Matrix metalloproteinase‐1 (MMP‐1) colorimetric drug discovery kit Anti‐Inflammatory Activity by measuring the nitric oxide production in the supernatant of the NHEK cells treated with lipopolysaccharide (LPS) Anti‐Tyrosinase Activity by described method in literature	[84]
*Oryza sativa* var (thai red rice)	Red rice stem cell cream showed superior anti‐aging effects than commercial cream. Volunteers experienced skin whitening, moisture increase, and enhanced elasticity.	Total phenolic and amino acid content using specific chemical assays Clinical irritation and anti‐aging effects on twenty‐eight healthy volunteers to determine melanin content reduction and skin elasticity	[89]
Apple of Sodom (Calatropisprocera) + Dead Sea Water (DSW) Extract	*C. procera* extract protects against skin irritation and inflammation biochemically. Combination with Dead Sea water enhances energy production and the extracellular matrix balance. *C. procera* extract shows no effect on cell viability and apoptosis.	Cytotoxicity by MTT assay test Cell Apoptosis by Caspase 3 Activity assay Anti‐Inflammatory Activity by measuring the nitric oxide production in treated ex vivo human skin organ culture (HSOC) with lipopolysaccharide (LPS) Anti‐Irritation Activity in treated exvivo human skin organ culture (HSOC) with Sodium Dodecyl Sulfate (SDS) Cytokines quantification analysis of human skin organ culture (HSOC) supernatants with specific ELISA kits for TNF*α*, IL‐1*α*and IL‐1*β*(Biolegend) and for Prostaglandin E 2 (PGE2, Enzo Life Sciences) Full thickness skin equivalents tested for gene expression and markers Protein biomarkers analyzed using immunoblotting and specific antibodies Gene expression screening and analysis for biological processes prediction	[90]
*Hibiscus sabdariffa* L. (Roselle)	Heat extraction for plant leaves and cells to obtain extracts. *Hibiscus sabdariffa* callus extract (HSCE) has anti‐melanogenic effects and skin barrier functions. *Hibiscus sabdariffa* plant extract (HSPE) up‐regulates genes in angiogenesis and glycolysis. HSCE up‐regulates ribosome proteins and IFI6 for skin healing. HSCE showed anti‐melanogenic effects and antioxidant activity. HSPE had more differentially expressed RNAs compared to HSCE.	HPLC Analysis of Water Extracts from *Hibiscus sabdariffa* Assessment of HSCE Cell Cytotoxicity Using the CCK‐8 Assayon human skin cell (HaCaT) Quantification of Melanin Content following HSCE Treatment B16F1 cells (mouse melanoma cell line) Expression Analysis of FLG and SOD1 Marker Genes by Real‐Time Reverse Transcription (RT‐PCR) on HaCaT cells Functional Characterization of Differentially Expressed Genes (DEGs) by Gene Ontology (GO) Enrichment Analysis Transcriptome profiling of human skin cells treated with extracts	[91]
*Chaenomeles japonica* (Thunb.) Lindl. ex Spach	Callus extract increased fibroblast proliferation rate significantly compared to control group. Fibroblasts treated with extract showed dose‐dependent morphology changes. Kinetin did not affect fibroblast proliferation rate in this study (Kinetin known from the literature as a small‐molecule plant compound that acts as a growth factor, that anti‐aging effects on cultured human skin cells have been reported).	Phytochemical analysis using UHPLC‐DAD‐ESI‐MS for compound determination. Antioxidant Activity: DPPH, FRAP, CUPRAC Assays Total Polyphenolic, Phenolic Acid, and Flavonoid Contents Cell proliferation ratio analysis on human skin fibroblasts with xCELLigence RTCA DP system	[92]
Tiarella polyphylla D. Don	*T. polyphylla* extract reduced MMP‐1 and increased type I procollagen. Callus extract protected against UVB toxicity, increasing cell viability and showed promising anti photoaging effects.	HPLC‐MWD for phytochemical profile analysis Cytotoxicity by MTT assay test on human fibroblasts (Hs68 cells) Cell Apoptosis by Caspase 3 Activity assay Anti‐Collagenase Activity on UVB treated Hs68 cells by Measurement of Type I Procollagen and MMP‐1 by ELISA kit Analysis of COL (1 and 3) Degradation and MMP (1–3, and) Expression in Hs68 Cells	[93]
*Juniperus communis* + German chamomile (Matricariarecucita) Processing Waste Extract	Combined extract showed higher sugar content, phenolics, and flavonoids. Chamomile extract had fatty acyls crucial for skincare products. Combination extract showed antioxidant, anti‐inflammatory, and antimicrobial properties. Extract combination was safe with no cytotoxic or phototoxic effects.	Cytotoxicity test using embryonal fibroblast cell line BALB/c 3 T3 from an albino laboratory‐bred strain from a house mouse and a phototoxicity test in human keratinocyte line HaCaT Chemical Characterization of Individual Extracts and Extract Combination total phenolic, flavonoid, tannin, and sugar contents Anti‐oxidant effect: DPPH radical scavenging assay Flow Cytometric Quantification—In Vitro Antioxidative Activity—the accumulation of reactive oxygen species (ROS) in UV‐irradiated HaCaT keratinocytes Analysis of the extract combinations effect on the accumulation of reactive oxygen species (ROS) in UV‐irradiated HaCaT keratinocytes reveal Flow Cytometric Quantification A real‐time cell monitoring system to proliferation of keratinocytes with regulating collagen type I and matrix metalloproteinase 1 (MMP‐1) production	[94]
*Centella asiatica* (L.)	Callus extract showed promising anti‐skin‐aging and antioxidant activities. Centella extracts exhibited potent radical scavenging properties without major flavonoids. callus extract (CE) and authentic plant extract (APE) enhanced fibroblast antioxidant enzyme expression differently. Callus extract showed antioxidant and anti‐skin‐aging activities on fibroblasts. Centella extracts prevented oxidative damage and maintained ATP levels in fibroblasts.	Cytotoxicity (Cell viability) by MTT assay test HPTLC‐DPPH and HPLC analysis for antioxidant compounds in extracts Oxidative stress treatment test with H_2_O_2_ on Human foreskin fibroblasts (BJ) Real time‐quantitative polymerase chain reaction (RT‐qPCR) for Effects of Centella extracts on expression of matrix metalloprotease 9 (MMP‐9).	[95]
Isodonrugosus (Wall. ex Benth.) Codd	Stem‐derived callus culture displays maximum total phenolic content and antioxidant activity. Rosmarinic acid (RA) was main contributor to antioxidant and anti‐aging activities. Pentacyclic triterpenoids correlated with elastase, collagenase, and tyrosinase inhibitions. *I. rugosus* calli produce antioxidant and anti‐aging bioactive extracts for cosmetics. *I. rugosus* in vitro cultures are a potential source for bioactive compounds.	Determination of Total Phenolic Compounds Content by HPLC Antioxidant effect: DPPH Assay Oxygen radical absorbance capacity (ORAC) Assay ABTS Assay Ferric reducing antioxidant power (FRAP) Assay Cupric ion reducing antioxidant capacity (CUPRAC) Assay Metal Chelating Activity Assay. Collagenase Assay (Matrix Metalloproteinase type 1 MMP1) Elastase Assay, Hyaluronidase Assay, Tyrosinase Assay, Anti‐AGE Formation Activity, SIRT‐1 Assay.	[96]
*Pyrus pyrifolia*	Callus extract promoted cell proliferation, wound recovery, and skin permeability. Extract exhibited high antioxidant activity comparable to ascorbic acid. and increased procollagen type I levels. Elasticnanoliposomes (NLs) loaded with extract have potential for skin rejuvenation. Callus extract promoted skin cell proliferation and wound recovery significantly. Extract exhibited Elastic NLs loaded with extract have potential as antiaging ingredients.	Antioxidant effect: DPPH Radical Scavenging Assay BTS Radical Scavenging Assay FRAP Assay Protein Glycation Assay Cell Proliferation Assay on keratinocyte (HaCaT) and fibroblast (CCD‐986sk) cell Antiwrinkle activity by Procollagen Synthesis Assay on fibroblast (CCD‐986sk) cell In Vitro Scratch Wound Recovery Assay on keratinocyte (HaCaT) and fibroblast (CCD‐986sk) cell In Vitro Skin Permeability on artificial skin	[97]
*Pyrus pyrifolia* var culta	Callus extract promotes skin regeneration, lightening, and anti‐aging activities. Extract exhibits high antioxidant activity (equivalent to 500 μM ascorbic acid) and inhibits melanogenesis (1.4‐fold reduction in melanocyte melanin compared to arbutin solution) effectively. Enhances fibroblast cell proliferation and procollagen synthesis significantly.	Major chemicalconstituents' identification including total phenolic and flavonoid content Evaluation of antioxidant activity using DPPH free radical‐scavenging assay Inhibition of tyrosinase activity and DOPA oxidation on mushroom tyrosinase activity Assessment of skin pigmentation inhibition by measuring melanogenesis of melanoma cells Cell proliferating assay on human fibroblast (CCD‐986sk) cells Evaluation of skin‐regenerating efficacy by promoting procollagen synthesis by ELISA on CCD‐986sk cells In vitro scratch wound recovery assay on CCD‐986sk cells	[98]
Citrus junos Siebold ex. Tanaka	Citrus junos callus extract inhibits tyrosinase activity and melanin biosynthesis. Callus extract reduced melanin content by 1.85‐fold in melanocytes. Callus extract promotes fibroblast proliferation and procollagen synthesis significantly. Citrus junos callus extract‐loaded NLs have anti‐aging and skin‐lightening effects. The callus extract accelerates scratch wound recovery in vitro.	Antioxidant effect: Determination of Antioxidant Capacity by the total phenolic content, The total flavonoid content by HPLC DPPH assay Skin‐Lightening Activity by Inhibition of Tyrosinase Activity (Inhibition of L‐DOPA Oxidation) and Melanin Biosynthesis in melanocytes (B16F10 melanoma cells). Skin Regeneration Activity by Effects of the Extract on Fibroblast Proliferation and Procollagen Synthesis and In Vitro Scratch Wound Recovery Effect of Citrus junos Callus Extract. In Vitro Skin Permeabilityacross and artificial skin and a human epidermal layer.	[99]
*Malus domestica* (apple) fruit callus	Apple callus exosome‐like nanovesicles (ACELNs) promoted collagen biosynthesis in UVA‐irradiated HDF cells. ACELNs showed concentration‐dependent increase in HDF and HaCaT cells. ACELNs did not induce cytotoxicity in HDF and HaCaT cells. ACELNs showed potential to prevent skin aging and enhance skin barrier.	Cytotoxicity inhibition assay with CCK‐8 kit and cell proliferation test by lactate dehydrogenase assay (LDH assay) on human dermal fibroblast (HDF) andhuman kerationocyte cell line (HaCaT). mRNA level Anti‐aging assay by qRT‐PCR on COL1A1 and FBN1 (Collagen and fibrillin as end‐product). Collagen synthesis analysis by COLIA1 kit on UVA‐irradiated HDF cells.	[100]
Woodfordiafruticosa Kurz.	WSC‐3 (callus extract of third month) WSC‐3 (callus extract of third month) increased collagen production by 60.67% in human dermal fibroblasts. WSC‐3 inhibited H_2_O_2_‐induced cellular senescence in HDF. Callus extract is non‐toxic (IC50: *N* 1000 μg/mL) in HDF. Meristem callus extract can substitute flower extract in skin care formulations.	LC–MS analysis conducted to identify phytoconstituents in callus extract. Cytotoxicity MTT assay in Human dermal fibroblasts (HDF). In vitro skin irritation potential by NRU assay in Mouse embryonic fibroblast (NIH3T3) cells Figure. Collagen induction rate by ELISA for collagen‐I on HDF cell.s Collagen‐I and elastin gene expression. Cellular senescence assay with H_2_O_2_ treatment and microscopical analysis.	[101]
Aster yomena	*A. yomena* callus extract alleviated UVB‐induced cell proliferation suppression and induced collagen formation. Callus extract reduced MMP‐1 expression and inhibited elastase in UVB‐irradiated cells. Callus extract improved skin damage recovery, moisture retention, and hyper‐keratinization. Callus extract reduced ROS, pro‐inflammatory cytokines, and MMPs in UVB‐irradiated cells. *A. yomena* callus extract can be used as a cosmetic ingredient for skin diseases.	UPLC/Q‐TOF‐MS for metabolite identification from callus extract qRT‐PCR for gene expression analysis in UVB‐irradiated HaCaT cells Cell Viability test on UVB irradiated HaCaT cells Antioxidant effect: DPPH Radical Scavenging Assay Measurement of Intracellular ROS Level by flow cytometer Measurement of Intracellular Nrf2 and NF‐κB Signals by flow cytometer Type I procollagen, matrix metalloproteinase‐1 (MMP‐1), tumor necrosis factor alpha (TNF‐*α*), IL‐8, and IL‐1*β* with specific ELISA kit Elastase activity test using N‐succinyl‐tri‐alanyl‐p‐nitroanilide (N‐STANA, elastase substrate)	[102]
Linumusitatissimum L.	(Neo)lignans accumulation in Linumusitatissimum varied under different light conditions. Flax lignans showed antioxidant and anti‐aging properties in vitro. Enterolignans from flax may reduce cancer risk post‐consumption. Lignans like epipinoresinol, secoisolariciresinol, and pinoresinol showed high correlations. Higher metabolite accumulation observed in cultures under light conditions. Antioxidant and anti‐aging properties were influenced by in vitro system and light.	Antioxidant activity: DPPH Radical Scavenging Assay ABTS Radical Scavenging Assay FRAP Assay CUPRAC Assay Collagenase Assay Elastase Assay Hyaluronidase Assay Tyrosinase Assay	[103]
*M. domestica* × *M. sylvestris* (Granny smith apple variety)	HPLC quantification revealed the presence of various phenolic compounds. Cell culture extract exhibited anti‐tyrosinase activity with high inhibitory effects. Callus and cell cultures produced phenolic compounds with anti‐tyrosinase activity. Gallic acid, rutin, and ferulic acid identified as major phenolic content.	HPLC‐based quantification of culture to quantify and identify phenolic constituents Cell viability determined using fluorescein diacetate (FDA) assay Evaluation of anti‐tyrosinase activity of cell culture extract on mushroom Tyrosinase activity	[104]

## 7 Challenges to Scalable Translation

Despite the potential outlined, several practical obstacles limit the transformation of callus culture into scalable, reproducible cosmetic products. First, **batch‐to‐batch consistency and genetic stability** remains a dominant concern: prolonged subculture can lead to somaclonal variation, in which karyotypic and epigenetic changes will alter the metabolite profile of a line in the long term. Routine cell‐bank management, well‐defined passage limits, and periodic genetic monitoring (e.g., flow‐cytometric ploidy checks or molecular markers) are needed to ensure that a production line continues to deliver the same active agent. Second, **standardization of metabolite fingerprints** is required so that each batch can be qualified against a reference profile; chromatographic and spectrometric methods (HPLC, HPTLC, LC–MS/MS) with one or more marker compounds and acceptance criteria provide the basis for such release specifications, but these are inconsistently reported in the literature. Third, the relatively **low metabolite yields** and the tendency of elicitation strategies to depress biomass (as seen in the jasmonic‐acid studies discussed above) create a yield–productivity trade‐off that must be optimized for each species before scale‐up in bioreactors. Finally, **contamination control and regulatory documentation** are the fundamentals for any cosmeceutical claim: sterile process design, microbial and endotoxin monitoring, and a documented quality report (including identity, purity, stability, and safety data) are needed to satisfy regulators and customers. Addressing these points would allow this field to function as a realistic manufacturing roadmap rather than a purely optimistic narrative.

## 8 Formulation, Stabilization, and Skin Delivery

Even a well‐characterized and highly effective callus extract can fail in practice if it is unstable in the formulation or cannot reach its site of action. Many of the relevant actives—polyphenols, flavonoids, and anthraquinones—are susceptible to oxidation, pH‐dependent degradation, and photodegradation. It should also be considered that several candidates are too hydrophilic or too large to cross the stratum corneum efficiently. Practical stabilization strategies include encapsulation in lipid‐based carriers such as solid lipid nanoparticles (SLNs) and nanostructured lipid carriers (NLCs), liposomes or elastic nanoliposomes (already applied to *Pyrus pyrifolia* and *Citrus junos* callus extracts), inclusion of co‐antioxidants and chelators to limit oxidation, and control of formulation pH and packaging (opaque, airless) to limit photo‐ and oxidative degradation. Skin permeability and the effect of these delivery systems should be evaluated rather than assumed: in vitro Franz diffusion cell studies (with synthetic membranes or excised skin), ex vivo human or porcine skin permeation, reconstructed human epidermis models, and tape‐stripping to quantify stratum corneum deposition are standard tools, ideally paired with analytical quantification of the marker compound recovered in each skin compartment. Reporting these parameters would substantially strengthen the “future trends” promise of this field and give formulation scientists actionable guidance.

## 9 Toward a Minimal Standard Panel for Callus‐Culture Anti‐Aging Studies

A recurring limitation across the studies reviewed here is heterogeneity in what is measured and how it is reported, which prevents direct comparison between extracts and species. To help the field move toward comparable, high‐quality evidence, we propose a minimal standard panel of assays and reporting items spanning the full development pipeline (Table 2). The goal is not to mandate every assay for every study, but to define a common backbone—metabolite profiling, stability, cell‐based efficacy endpoints, penetration testing, safety, and human outcomes—together with the contextual parameters (callus line, induction and elicitation conditions, extract type, dose, and positive controls) needed to interpret and reproduce a result.

## 10 Conclusion

The exploration of callus culture technology signifies a transformative advancement in the domain of skin antiaging research, offering a sustainable and efficient mechanism for the production of bioactive compounds endowed with potent geroprotective properties. This sophisticated methodology facilitates the controlled biosynthesis of valuable phytochemicals while mitigating the inherent variability and contamination risks associated with traditional plant sourcing, thus ensuring a consistent and reliable supply of botanical ingredients for the health and cosmetic industries. The strategic manipulation of phytohormonal pathways within callus culture not only enhances the yield of secondary metabolites with therapeutic and cosmetic efficacy but also adheres to the ethical principles of environmental stewardship. Furthermore, the burgeoning consumer demand for natural ingredients has led to a market shift toward the use of botanical preparations for antiaging applications. Investigations into various plant species, such as *Malus domestica* (apple), *Oryza sativa* (rice), *Centella asiatica* , and *Aster yomena*, have revealed that callus cultures can generate phytochemicals rich in antioxidants and anti‐inflammatory agents, which are instrumental in promoting skin health and alleviating oxidative stress, thereby preventing cutaneous senescence. These in vitro cultures have demonstrated remarkable efficacy in enhancing keratinocyte proliferation, stimulating collagen synthesis, inhibiting elastase activity, and diminishing inflammatory markers associated with skin aging. Consequently, the extracts derived from these callus cultures serve as invaluable sources of geroprotective agents for cosmeceutical and pharmaceutical applications. The integration of callus culture techniques not only fosters the exploration of bioactive compounds but also paves the way for the development of innovative formulations that address various skin concerns, including wrinkles and loss of elasticity. Ultimately, the incorporation of callus culture into skin antiaging research heralds a significant breakthrough, promising the creation of effective antiaging skincare products that are both environmentally friendly and aligned with consumer preferences. At the same time, while mentioning the potential, current limits of the evidence should be acknowledged. Most reported effects rest on acellular antioxidant assays and cell‐based models, while controlled human data remain rare; the range of species studied is narrow, methods are not standardized, and formal safety and regulatory documentation is largely absent. The future research priorities therefore are: (i) larger, controlled human studies using instrumented, standardized endpoints (such as wrinkle scores, elasticity, hydration/TEWL, and pigmentation); (ii) systematic safety testing, including sensitization and irritation; (iii) standardized metabolite fingerprinting and manufacturing controls that will ensure batch consistency and genetic stability; and (iv) clear regulatory pathways for cosmeceutical claims. Adoption of the minimal reporting panel proposed in Table 2 would make future studies directly comparable. Addressing these gaps will be essential before callus‐culture‐derived actives can be considered, with confidence, as evidence‐based anti‐aging ingredients rather than promising candidates.

## Author Contributions

**Amir Mohammad Sharafi**, **Ali Khajeei** and **Tooba Gholikhani** conceptualized the study. **Amir Mohammad Sharafi**, **Maryam Hasan Zadeh Navroodi**, **Seydeh Halimeh Najafi**, and **Tooba Gholikhani** developed the methodology. **Amir Mohammad Sharafi**, **Ali Khajeei**, **Mohaddeseh Argha**, **Faezeh Talaei Shahmirzadi**, and **Tooba Gholikhani** conducted the investigation. **Amir Mohammad Sharafi**, **Ali Khajeei**, **Seydeh Halimeh Najafi**, and **Tooba Gholikhani** drafted the original manuscript. **Amir Mohammad Sharafi**, **Ali Khajeei**, **Soheila Mokari**, and **Tooba Gholikhani** reviewed and edited the manuscript. **Amir Mohammad Sharafi**, **Parina Asgharian**, and **Tooba Gholikhani** supervised the project.

## Ethics Statement

The authors have nothing to report.

## Consent

The authors have nothing to report.

## Conflicts of Interest

The authors declare no conflicts of interest.

## Data Availability

The data that support the findings of this study are available from the corresponding author upon reasonable request.
